# Highlights of ASCO 2026

**DOI:** 10.1093/oncolo/oyag354

**Published:** 2026-09-03

**Authors:** Brandon A DaSilva, Alfred I Neugut, Tito Fojo, Susan E Bates

**Affiliations:** Department of Medicine and Herbert Irving Comprehensive Cancer Center, Columbia University, New York, NY 10032, United States; Vagelos College of Physicians & Surgeons, and the Department of Epidemiology, Columbia University, New York, NY 10032, United States; Department of Medicine and Herbert Irving Comprehensive Cancer Center, Columbia University, New York, NY 10032, United States; Vagelos College of Physicians & Surgeons, and the Department of Epidemiology, Columbia University, New York, NY 10032, United States; Mailman School of Public Health, Columbia University, New York, NY 10032, United States; Department of Medicine and Herbert Irving Comprehensive Cancer Center, Columbia University, New York, NY 10032, United States; Vagelos College of Physicians & Surgeons, and the Department of Epidemiology, Columbia University, New York, NY 10032, United States; James J. Peters Bronx VA Medical Center, Bronx, NY 10468, United States; Department of Medicine and Herbert Irving Comprehensive Cancer Center, Columbia University, New York, NY 10032, United States; Vagelos College of Physicians & Surgeons, and the Department of Epidemiology, Columbia University, New York, NY 10032, United States; James J. Peters Bronx VA Medical Center, Bronx, NY 10468, United States

Once again this year, we present highlights from the annual meeting of the American Society of Clinical Oncology, held in Chicago, with over 40 000 attendees and over 5000 presentations. Many studies were interesting for a variety of reasons, so we had to choose carefully for the readers of *The Oncologist*. The abstracts can be accessed at ASCO.org; citations for the concurrent publication were included where available. It was a good year for gastrointestinal cancers.

## Gastrointestinal cancers

- Targeted therapies, often eclipsed by immunotherapy over the past decade, re-emerged as a focal point. The most talked about study was presented in the Plenary Session (Abstract LBA5) by Brian Wolpin of Dana-Farber Cancer Institute in Boston MA. The excitement generated by this study derived from unprecedented results with an oral agent for advanced pancreatic adenocarcinoma (PDAC). RAS mutations are found in 90%-95% of PDACs. Daraxonrasib is an investigational oral pan-RAS(ON) inhibitor that targets a broad spectrum of active RAS mutants in solid tumors as well as wild-type RAS variants while ignoring the inactive “OFF” state. We all recognize the lethality of this disease following progression after first-line chemotherapy. In the RASolute 302 phase 3 trial, 500 patients with metastatic PDAC that had progressed following first-line therapy were randomized at 59 global sites to either daraxonrasib 300 mg daily or investigator’s choice of chemotherapy.^1^^,^^2^ The overall response rate was 31.6% with daraxonrasib vs 11.2% with investigator choice chemotherapy. The most impactful adverse effects recorded with daraxonrasib were rash, stomatitis, diarrhea, nausea and vomiting. With a median follow-up time of 8.5 months, the median overall survival (OS) was 13.2 months for daraxonrasib vs 6.7 months for chemotherapy (hazard ratio [HR] 0.40, 95% confidence interval [CI] 0.30-0.53). We recognize the relatively short follow-up was driven by the ASCO Annual Meeting timeline and appreciate OS as the appropriate endpoint for second-line pancreatic cancer. Although a KM-derived median OS can be reported when median follow-up is shorter than the estimated median survival, such an estimate should be interpreted cautiously. With a median follow-up of only 8.5 months, the median OS of 13.2 months was based on only a handful of observations/participants beyond the median and can be sensitive to censoring and the number of patients remaining at risk. A second area of concern is the notation that therapy was administered for a median 1.9 months in the control arm. This is much less than expected for patients fit enough to enroll on second-line clinical trials and suggests some patients may have left the study before progression due to disappointment with randomization to the control arm. The latter possibility is reinforced by the observation that 15% of those randomized to chemotherapy did not get any protocol chemotherapy. A concern in these latter features is the apparent loss of equipoise that underpins any randomized clinical trial. It was known that daraxonrasib provided meaningful tumor shrinkage and control since its evaluation in phase 1/2 studies.^3^ While we acknowledge its activity, we look forward to more mature data.
- Targeted therapies, often eclipsed by immunotherapy over the past decade, re-emerged as a focal point. In colorectal cancer, the BRAF V600E mutation, present in approximately 10% of malignancies, has particular significance because its presence generally heralds a worse outcome. Recently, the addition of encorafenib, a BRAF inhibitor, was shown to be effective in combination with cetuximab in treating this group of patients. This effectiveness is felt to occur because BRAF inhibition can lead to epidermal growth factor receptor (EGFR)-driven reactivation of the BRAF pathway with attenuation of the activity of BRAF inhibitors, and targeting BRAF simultaneously with EGFR inhibition can help overcome this reactivation.^4^ Previously, the addition of encorafenib and cetuximab to FOLFOX was shown in the BREAKWATER Trial to improve progression-free survival (PFS) and OS compared to standard therapy.^5^ In this year’s follow-up study, presented by Scott Kopetz of the MD Anderson Cancer Center, the investigators randomized 147 patients with measurable disease and metastatic colorectal cancer to encorafenib plus cetuximab with FOLFIRI or FOLFIRI with or without bevacizumab.^6^^,^^7^ The primary endpoint was objective response rate (ORR); this was 64.4% for the experimental group vs 39.2% for the reference cohort (*P *= .0011). At a median follow-up of 18.0 months, the median PFS was 15.2 months for the intervention arm vs 8.3 months for the control group (HR 0.44, 95% CI 0.27-0.70). At 18 months, the OS was 72.0% vs 54.5% (HR 0.56, 95% CI 0.34-0.94). Although the authors claimed the EC plus FOLFIRI regimen was “generally tolerable,” 10% discontinued encorafenib, 24% had a dose reduction, and 65% a dose interruption, guaranteeing utilization in the real-world will be difficult and require dose adjustments and likely not emulate the clinical trial results.
- For patients with unresectable or metastatic cholangiocarcinoma, the leading first-line regimen at present is gemcitabine/cisplatin with immunotherapy. However, in a striking resemblance to non-small cell lung cancer (NSCLC), various molecular targets have emerged as critical for oncogenesis, providing therapeutic options. One such molecular target is a FGFR2 rearrangement/gene fusion that is found as a putative oncogenic driver in approximately 10-15% of intrahepatic cholangiocarcinomas. The gene fusion results in ligand-independent constitutive dimerization and activation of the FGFR2 kinase. Pemigatinib inhibits ATP binding to the FGFR kinase domain, shutting down the constitutive signaling, resulting in response rates up to 40%. Although recognized as a key target, inhibition of FGFR2 is only FDA approved in the second line, after chemotherapy. The phase 3 FIGHT-302 trial, presented by Tanios Bekali-Saab of the Mayo Clinic in Phoenix AZ, randomized 167 patients with untreated metastatic cholangiocarcinoma bearing an FGFR2 rearrangement (Abstract 4017).^8^^,^^9^ The experimental arm received oral pemigatinib while the control group received gemcitabine plus cisplatin. The waterfall plots demonstrating a robust 47% response rate for pemigatinib compared to 15.5% for gemcitabine plus cisplatin provide clear evidence of activity. A claimed median duration of response of 14.2 months for pemigatinib compared to 6.3 months for gemcitabine plus cisplatin is similarly impressive. But unexplained is how, with a median follow-up of 42.1 months, these advantages generated only a 1.5 month PFS advantage (8.34 months for pemigatinib and 6.80 months for the chemotherapy [HR 0.584, 95% CI 0.393-0.868]). That such a small PFS difference, driven in part by early differential censoring, disappeared by the time of the OS analysis with medians of 24.4 vs 25.0 months for pemigatinib vs gemcitabine plus cisplatin (NS) is not surprising. But compared to median values for the TOPAZ and Keynote 966 studies of 12.8 and 12.7 months with immunotherapy, respectively, the duration of OS for both arms was also puzzling.^10^^,^^11^ While crossover would eventually be allowed, only 35% of the chemotherapy arm received pemigatinib. As regards those randomized to pemigatinib, we know very little of what they may have received post-trial. Having experienced disease progression within 8 months, it is hard to believe they would have survived an additional 16 months without further treatment. We thus walk away convinced pemigatinib is an active agent, but uncertain how this can be translated to better survival outcomes.
- When a study question is important, even negative/null findings deserve dissemination. ATTRACTION-6 was a phase 3 trial conducted between 2021 and 2023 in South Korea, Japan, and Taiwan adding ipilimumab to nivolumab in yet another disease without improving outcomes, but exacerbating toxicties.^12^ It was designed to assess the addition of nivolumab and ipilimumab to first-line chemotherapy for patients with advanced HER2-negative gastric/gastroesophageal cancer. The results were presented by Do-Youn Oh of Seoul National University College of Medicine in Seoul, South Korea (Abstract 4006). In the study, 626 patients were randomized to nivolumab (360 mg every 3 weeks) plus ipilimumab (1 mg/kg every 6 weeks) with chemotherapy (*n* = 315) or chemotherapy alone (*n* = 311). Chemotherapy consisted of either capecitabine plus oxaliplatin or S-1 plus oxaliplatin. At a median follow-up of 31.3 months, the median OS was 15.7 months for the experimental arm vs 15.8 months for the control arm (HR 0.90, 95% CI 0.74-1.09). The median PFS was 8.9 months for the experimental arm vs 7.7 months for the control arm (HR 0.83, 95% CI 0.69-1.00). The study investigators concluded that the addition of ipilimumab plus nivolumab did not improve outcomes. But why not, when as the presenter noted, ICI plus chemotherapy is an established first-line option for HER-2 negative advanced G/GEJ cancer with OS benefit *especially on PD-L1 positive tumors*? The answer may be multifactorial and include PD-L1 expression and efficacy/tolerability/drug dosing. Previously, Checkmate-649 achieved an OS benefit in a study population where 60% of patients had tumors with PD-L1 levels ≥5 and 81% had PD-L1 levels ≥1.By comparison, no OS benefit was found in ATTRACTION-6,where only 44.4% of tumors had PD-L1 levels ≥5 nor the earlier ATTRACTION-4 study in which only 16% had PD-L1 levels ≥1.^12–14^ Early gains did not improve with time and the “sustained separation” of the curves noted by the presenter, are a violation of the proportional hazards assumption suggesting, in fact, a worsening over time, possibly explained by compromising the chemotherapy backbone as suggested by the statement that “toxicity management is essential to maintain treatment intensity.” This would not be the first time the addition of a toxic adjunct compromised the essential chemotherapy backbone and, in turn, efficacy. We look forward to seeing more details on this study.

## Lung cancer

- Jonathan Goldman of UCLA in Los Angeles CA presented the findings of the phase 3 LIBRETTO-432 trial at the Plenary Session (Abstract LBA3).^15^^,^^16^ This study addressed the treatment of patients with early-stage RET fusion-positive NSCLC, a target that is present in 1%-2% of those with NSCLC. Selpercatinib is a RET kinase inhibitor that has proven efficacy in patients with *RET* fusion-positive Stage IB, II or IIIA NSCLC. One of its advantages is that it is CNS-penetrant. The LIBRETTO-432 trial enrolled patients who had received definitive surgery or radiotherapy ± systemic adjuvant chemotherapy and had no evidence of disease recurrence or progression following this definitive therapy—hence event-free survival (EFS) was the primary endpoint. The trial randomized 151 individuals with RET fusion-positive NSCLC from 22 countries to receive selpercatinib or placebo for three years. With a median follow-up of 24 months, the median EFS was not yet reached for those on selpercatinib, while the EFS for the placebo group was 31.8 months (HR 0.125, 95% CI 0.028-0.552). The EFS rate at 2 years was 91.5% for selpercatinib vs 61.1% for placebo. Selpercatinib did not formally go through the FDA’s Project Optimus because its initial development and accelerated approval occurred before the project was launched in 2021. However, it is alleged its clinical trial design used the exact dose-optimization strategies that Project Optimus now requires. We hope not. Why? Because 77.3% of patients had dose interruptions due to TEAEs; 54.7% had dose reductions due to AEs; and 17.3% discontinued treatment due to TEAEs. We would argue this is a miserable track record for a drug we want to give for 3 years, or longer. Hepatotoxicity was a major problem and, according to NIH LiverTox data, liver damage with selpercatinib is not caused by off-target blocking of a critical liver kinase, but rather it stems from low-level direct hepatocellular injury following liver metabolism by CYP-3A4. The accumulation of the drug and its metabolites create localized cellular stress triggering direct hepatocyte inflammation. The bottom line is that we need more specific/less toxic inhibitors or results with better tolerated selpercatinib doses that can be emulated in the real-world.
- Precision medicine and biomarkers continue to make important inroads into the treatment of NSCLC. We witnessed a new successful treatment for a targetable genetic aberration, EGFR exon 20 insertion mutations, which have evaded the treatment effects of other EGFR targeting therapies like osimertinib. These mutations are found in 12% of EGFR-mutated NSCLC which represent approximately 2%-3% of overall NSCLC. Abstract LBA8500 was presented by John Heymach of the University of Texas MD Anderson Cancer Center in Houston TX, encompassing the results of the WU-KONG28 trial.^17^^,^^18^ In this trial, 324 patients with advanced non-squamous NSCLC with EGFR exon 20 insertions were randomized to either sunvozertinib, an oral tyrosine kinase inhibitor, or to standard chemotherapy with pemetrexed and carboplatin. With a median follow-up time of 12 months, the ORR was 58.9% vs 31.2% for chemotherapy. The median PFS was 10.3 months for sunvozertinib vs 7.5 months for the platinum-based chemotherapy (*P* < .001). Promising results for which we await the OS result that was not yet available.
- Ivonescimab is a bispecific antibody that targets two antigens simultaneously, VEGF and PD-1, thereby simultaneously blocking blood vessel formation and enhancing the immune response. Shun Lu of the Shanghai Lung Cancer Center at Shanghai Chest Hospital in Shanghai, China, presented the results of the HARMONi-6 phase 3 trial at the plenary session (Abstract LBA4).^19^^,^^20^ The study randomized 532 people with untreated advanced (stages IIIB, IIIC, or IV) squamous cell carcinoma of the lung to receive either ivonescimab in combination with paclitaxel + carboplatin or the anti-PD-1 antibody tislelizumab with paclitaxel + carboplatin. In both arms of the trial, the patients received the regimen with chemotherapy every 3 weeks for 4 cycles and then continued either ivonescimab or tislelizumab monotherapy for up to 24 months. With a median follow-up of 21.4 months, the OS was 27.9 months for the ivonescimab group vs 23.7 months for tislelizumab (HR 0.66, 95%CI 0.50-0.87). Conceptually, ivonescimab is designed to consolidate and replace the therapeutic strategy of combining a PD-L1 inhibitor with a VEGF inhibitor, such as the combination of atezolizumab and bevacizumab. It is a single engineered bispecific antibody rather than two separate drugs. But rather than just serving as a convenient “two-in-one” option, it seeks to leverage the chemistry of cooperative binding with a distinct tetravalent structure that triggers a localized domino effect in the tumor microenvironment. More importantly, it seeks to overcome the “squamous NSCLC safety barrier,” and early data suggest that it may be succeeding given it does not appear to increase the risk of severe bleeding complications that plagued bevacizumab in squamous lung cancers. The bispecifics have arrived and ivonescimab is increasingly making a case for its use as a standard of care therapy.

## Genitourinary cancers

### Prostate cancer

- In an innovative approach to the management of high-risk localized prostate cancer or locally advanced prostate cancer (HR LPC/LAPC), Mary-Ellin Taplin from Dana-Farber Cancer Institute presented the PROTEUS study (Abstract LBA1).^21^^,^^22^ This study investigated the effects of perioperative androgen deprivation therapy (ADT) + apalutamide vs ADT + placebo in patients planned for a radical prostatectomy with pelvic lymph node dissection. In total, 2109 patients were randomized, with 1057 patients receiving ADT + apalutamide and 1052 receiving ADT + placebo for 6 cycles prior to and 6 cycles following radical prostatectomy. There were two primary endpoints—composite pathologic CR or minimal residual disease (pCR/MRD), and metastasis-free survival (MFS) based on PSMA PET. With a median follow-up of 61.7 months, ADT + apalutamide led to higher, albeit modest rate of pCR/MRD compared to ADT + placebo (8.9% vs. 1%, odds ratio (OR) 10.17, 95%CI 5.27-19.64) and 5-year MFS rate of 78.2% vs 73.5%, respectively.
- Median EFS was improved with perioperative ADT + apalutamide (57.1 months) compared to ADT + placebo (38.4 months) (HR 0.71, 95% CI 0.63-0.80). PROTEUS lacks the necessary controls—ADT + apalutamide either pre- or post-operative alone, important given a 27.5% incidence of treatment-related grade 3 or 4 serious AEs (SAEs) and a 3.5-fold greater likelihood of those AEs leading to dose reduction or interruption. Nor do we know about the all-important OS that would likely be similar after salvaging the reference arm with abiraterone, enzalutamide, apalutamide, or darolutamide.^23^

## Breast cancer

- Sara Hurvitz from the Fred Hutchinson Cancer Center in Seattle, Washington presented the findings of the phase 3 VIKTORIA-1 Study 2 **(**Abstract LBA1008**)** investigating the effects of the pan-PI3K and mTORC1/2 inhibitor gedatolisib with fulvestrant and palbociclib (GED, GED triplet) vs gedatolisib + fulvestrant (GED doublet) vs alpelisib + fulvestrant in patients with hormone receptor (HR) positive, HER2-negative advanced breast cancer with PIK3CA mutations whose disease had progressed on a CDK4/6 inhibitor and an aromatase inhibitor.^24^ In total, 362 patients were randomized to either the GED triplet arm (155 patients), alpelisib + fulvestrant (155 patients), or the GED doublet arm (52 patients). With a median follow-up of 11 months, the median PFS was 5.6 months for alpelisib + fulvestrant compared to 11.1 months for GED triplet. (HR 0.50, 95%CI, 0.37-0.68), and a similar 11.3 months for the GED doublet (HR 0.51, 95% CI, 0.33-0.79). Important OS data were not yet mature. A closer look at the data reveals almost certain toxicities that will emerge as challenging and practitioners will have to watch for in considering any such regimens. The GED triplet has the highest response rate at 65% compared to 35% for the doublet, yet the median duration of response (DoR) in the GED triplet only 15.7 months compared to 24.7 months in the GED doublet arm. This somewhat unexpected observation is likely due to the added toxicity of the GED triplet which with palbociclib a known marrow suppressant had a 63.4% all grades neutropenia, including 47.7% G3 and 11.1% G4, toxicity grades that likely led to dose reductions and delays and saw a higher response rate followed by a shorter DOR. Likewise although hyperglycemia, a known class effect of PIK3CA inhibitors was seen in 57.9% of patients in the alpelisib + fulvestrant arm with grade 3 events in 13.8%, the frequency of all grade treatment-related hyperglycemia in patients exposed to gedatolisib was 15% and 11.5% for the GED triplet and doublet, respectively, with grade 3 events in 2.6% and 0% of patients. However, the incidence of stomatitis was 61.4% and 61.5% in the GED doublet and triplet, respectively, with the higher rate of stomatitis with gedatolisib compared to alpelisib primarily driven by gedatolisib’s concurrent inhibition of the mammalian target of rapamycin (mTOR) pathway. The extent to which all of this will contribute to efficacy and tolerability will now be in the hands of practitioners. But one can be concerned that the approved 180 mg dose of gedatolisib as an intravenous infusion over 30 minutes once weekly on days 1, 8, and 15 of every 28 day cycle may not be tolerable. We note that although of the 129 patients who discontinued the GED triplet, only 4 were ascribed to an adverse event, 29/109 or 27% discontinued for either adverse event, “patient decision and investigator decision” the latter often surrogates of poor tolerability, comparted to 19.8% of those assigned to alpelisib plus fulvestrant. Finally, note there were two VIKTORIA studies. Study 1 (PIK3CA-WT disease) completed enrollment ahead of Study 2 (PIK3CA-mutated disease), and on July 14.2026 the USFDA approved gedatolisib in combination with fulvestrant with or without palbociclib specifically thus far for patients whose tumors DO NOT HAVE a PIK3CA mutation.
- Challenging the standard of TCHP (docetaxel [T], carboplatin [C], trastuzumab [H], and pertuzumab [P]) in the neoadjuvant setting for HER2+ early or locally advanced breast cancers, Zhi-Ming Shao from Fudan University Shanghai Cancer Center in China shared their findings with anbenitamab, an antibody engineered to simultaneously target two different non-overlapping epitopes on Her2 (biparatropic) with Arm 1 targeting Domain II of Her2 and Arm 2 targeting Domain IV of Her2 (bispecific). In the randomized phase 3 Neo-Healer trial, anbenitamab was administered in combination with albumin-bound docetaxel (nab-docetaxel) ± carboplatin (anbenitamab arm) against the standard of care (TCHP).^25^ The primary endpoint was total pathologic CR (tpCR). Of 521 patients randomized, 263 were treated on the anbenitamab arm and 258 on the TCHP arm. The tpCR in the anebenitamab arm was 62.4% vs 51.2% in the TCHP arm (95%CI for difference, 3.19%-19.63%). The improvement in tpCR in the anbenitamab arm comes without added grade ≥3 TEAEs that occurred in 29.3% and 28.3% of patients, respectively, but with slightly higher all grade toxicities. Although it will take time for the data to mature, it will be intriguing to see whether these responses ultimately translate into event-free and OS benefits. In discussing ivonescimab, we noted the bispecifics have arrived, so too have the biparatropic bispecifics. The drug was approved in China in May 2026 and has been granted Orphan Drug Designation by the FDA for treatment of HER-2 positive gastric cancer per the KC-WISE trial.^26^

## Gynecologic oncology

### Ovarian cancer

- In Abstract 5503, Lucy Gilbert from McGill University Health Centre in Montreal, Canada reported subgroup analyses for the phase 3 ROSELLA trial investigating relacorilant + nab-paclitaxel vs nab-paclitaxel monotherapy in patients with platinum-resistant ovarian cancer (PROC).^27^^,^^28^ Relacorilant is a first-in-class, glucocorticoid receptor antagonist that sensitizes cancers to chemotherapy by disrupting cortisol-driven cancer cell survival. In this study, 381 patients were randomized to receive relacorilant on the day prior to, day of, and day after receiving nab-paclitaxel (188 patients) or nab-paclitaxel alone (193 patients). The PFS by BICR had previously been reported with a median PFS of 6.5 months in the relacorilant arm vs. 5.5 months in the nab-paclitaxel arm (HR 0.70, 95% CI 0.51-0.91). Although there appeared to be a minimal clinical benefit in PFS, the OS was extended by 4.1 months (16 vs 11.9 months; HR 0.65, 95% CI 0.51-0.83), with the magnitude increasing with time. Although limited by a smaller cohort size (22 patients and 33 patients, respectively), they reported that patients with shorter taxane-free intervals (≤6 months), still derived a survival benefit from the addition of relacorilant to nab-paclitaxel (median OS 16.7 vs 11.0 months; HR 0.60, 95% CI 0.31-1.15). This is compared to patients with longer taxane-free intervals (>6 months), where the result was statistically significant, but the absolute median OS difference was shorter (3.6 months), likely the result of larger subgroup sizes. Despite a reduction of the paclitaxel dose from 100 mg/m^2^ on days 1, 8 and 15 of a 21-day schedule in the reference arm to 80 mg/m^2^ in the experimental arm, the addition of relacorilant to nab-paclitaxel increased the frequency of grade ≥3 adverse events (74.5% vs 59.5%), driven by differences in neutropenia (44% vs 18%), anemia (25% vs 9%), and fatigue (9% vs 2%). Thus, the improvement in OS must be balanced against a clear increase in toxicity, likely due at least in part to a drug–drug interaction that resulted in an increase in drug exposure. Relacorilant is both a strong CYP3A inhibitor and a CYP3A substrate, while paclitaxel is metabolized heavily by both the CYP3A4 and CYP2C8 pathways. When relacorilant blocks CYP3A4, paclitaxel’s clearance via CYP3A is reduced, however, paclitaxel has a strong secondary clearance route through CYP2C8 and because relacorilant does not inhibit CYP2C8 *in vivo*, paclitaxel can still be safely eliminated by the liver, resulting in only modest, manageable increases in systemic paclitaxel exposure. The package insert cautions that care should be exercised in a patient taking a strong/moderate CYP3A inducer and CYPC28 inducers and inhibitors. It recommends monitoring for reduced effectiveness of relacorilant with nab-paclitaxel with CYP2C8 inducers and moderate CYP3A inducers, and an increase in the risk of adverse reactions with co-administration of CYP2C8 inhibitors since they may increase concentrations of paclitaxel. In a vulnerable patient population with median OS of ∼12 months, these data represent an initial positive step for patients with PROC and prior taxane exposure, but oncologists should proceed with care.

## Malignant hematology

- Abstract 7507 provided further evidence supporting the use of anti-BCMA x CD3 bispecific T-cell engager, teclistamab (TEC), in relapsed/refractory multiple myeloma (RRMM). Presented by Roberto Mina of Winship Cancer Institute at Emory University, the MajesTEC-9 trial investigated single-agent TEC vs investigator’s choice of either pomalidomide/bortezomib/dexamethasone (PVd) or carfilzomib/dexamethasone (Kd) in RRMM patients with 1-3 prior lines of therapy and exposure/refractoriness to an anti-CD38 antibody and lenalidomide.^29^^,^^30^ In this trial, 593 patients were randomized, with 296 receiving TEC and 297 receiving PVd/Kd. Approximately 74% of patients in each arm were considered double refractory to anti-CD38 therapy and lenalidomide. In the TEC arm, 65.8% achieved a CR compared to 16.8% in the PVd/Kd arm (OR 10.42; 95% CI 6.89-15.76). The 18-month PFS was 69.8% compared with 26.9% and 18-month OS was 79.2% vs 68.6% (HR 0.60; 95% CI, 0.43-0.83). The toxicity profile of TEC was favorable and aligned with known side effects. With respect to cytokine release syndrome (CRS) and immune effector cell associated neurotoxicity (ICANS), both were predominantly low-grade (grade 1/2) and there were no grade 4 events. All CRS events resolved without stopping TEC.

### Lymphoma, diffuse large B-cell lymphoma

- Results of the phase 3 international frontMIND study in newly diagnosed diffuse large B-cell lymphoma (DLBCL) or high-grade B-cell lymphoma (HGBL), Abstract LBA7000, were presented by Georg Lenz from University Hospital Munster in Germany.^31^^,^^32^ In a cohort of 899 patients, 448 patients were randomized to receive 6 cycles of the anti-CD19 monoclonal antibody Tafasitamab (Tafa) and immune-modulator lenalidomide (Len) plus R-CHOP vs 451 patients to receive 6 cycles of R-CHOP. With a median follow-up of 35.2 months, the primary endpoint of investigator-assessed PFS was met (HR 0.75; 95% CI, 0.59-0.96). The 2-year PFS was 71.1% in the Tafa-Len-R-CHOP group vs 62.9% with R-CHOP alone. Although longer follow-up for OS is needed, the benefit of adding Tafa-Len to R-CHOP over R-CHOP alone with respect to PFS has shown improvement in efficacy without compromising on added toxicity. In a population at high-risk for disease recurrence, this regimen potentially adds to the armamentarium of front-line options in DLBCL and HGBL.

## Circulating tumor DNA

- It is not an exaggeration to say that ctDNA testing has been one of the most revolutionary innovations in oncology in the past decade and we have not yet fully realized its benefits. Colon cancer has been the first major cancer to benefit from this technology, and with Abstract LBA3500, presented by Gunnar Folprecht of Technical University Dresden, Dresden, Germany, we learn the results of the CIRCULATE trial. In this trial, conducted in Germany and Austria, 1396 patients with MMR-proficient stage II colon cancer were screened with ctDNA ≥5 days after resection between June 2020 and July 2025.^33^^,^^34^ Of these, 41 (2.9%) proved positive. While adjuvant chemotherapy for stage II colon cancer is not routinely recommended, these patients are considered high risk and were randomized in a 2:1 ratio to either adjuvant chemotherapy (capecitabine ± oxaliplatin) or observation. The pre-specified analysis was at 3 years, at which time the recurrence rate was 62% for the ctDNA-positive patients on the observation arm vs 19% for those who had chemotherapy (HR 0.23, 95% CI 0.06-0.87). The 3-year DFS was 38% vs 77%, respectively (HR 0.31, 95% CI 0.09-.03). The authors concluded that this justified a ctDNA-guided treatment strategy for patients with stage II colon cancer. The discussant agreed, and prevailing opinion seems also to have shifted in the direction of treating ctDNA-positive stage II colon cancer.

## Non-chemotherapeutic interventions

- The benefits of Glucagon-like peptide-1 (GLP-1) receptor agonists have been widely studied in diabetes, cardiovascular disease, and kidney disease. This ASCO meeting featured several abstracts highlighting the effects of GLP-1 receptor agonists in cancer. In their poster, Abstract 3143, Mark Orland and colleagues at the Taussig Cancer institute at the Cleveland Clinic in Ohio performed a retrospective propensity score-matched analysis for patients with stages I-III cancers on GLP-1 receptor agonists and DPP-4 inhibitors across seven malignancies and assessed progression to metastatic disease.^35^ They found that GLP-1 agonist exposure was associated with statistically significant reductions in progression to metastatic disease in NSCLC, breast, colorectal, and hepatocellular carcinomas. There was a trend towards a protective benefit in prostate and pancreas cancers and no benefit in renal cell carcinoma. The authors also note that increased expression of GLP-1 receptors on tumors correlated with OS, with the most notable effect in breast cancer (HR 0.55, 95% CI 0.35-0.87).
